# Responsiveness of the CCVUQ-Br quality of life questionnaire in chronic venous ulcer patients

**DOI:** 10.1590/1677-5449.190047

**Published:** 2020-01-09

**Authors:** Renata Cardoso Couto, Flávia de Jesus Leal, Guilherme Benjamin Brandão Pitta, Solange Andreoni

**Affiliations:** 1 Universidade Federal de São Paulo – UNIFESP, São Paulo, SP, Brasil.; 2 Universidade Estadual de Ciências da Saúde de Alagoas – UNCISAL, Maceió, AL, Brasil.

**Keywords:** venous ulcer questionnaire, sensitivity and specificity, quality of life

## Abstract

**Background:**

Responsiveness is a measure of an instrument’s ability to reflect in its score the variability that has occurred in a patient’s life as a result of an intervention. The CCVUQ-Br has been validated in Portuguese, but its responsiveness still needs to be tested. When this study has been completed, the CCVUQ-Br will be available for use as an instrument capable of detecting and reflecting in its score the changes that take place in the quality of life of people with venous ulcers.

**Objectives:**

To evaluate the responsiveness of the CCVUQ-Br.

**Methods:**

A longitudinal intervention study was conducted at public and private centers for patients with venous ulcers. The sample comprised people with chronic venous ulcers due to start treatment and the variables analyzed were CCVUQ-Br score and its domain scores, a pain visual analog scale (pain VAS), and the Global Assessment of Change Scale, in addition to CEAP classification, and size of ulcer. The CCVUQ-Br was administered to 51 people about to start treatment who were recruited at random. The CCVUQ-Br was then re-administered 4 weeks after treatment had started.

**Results:**

Mean CCVUQ-Br scores reduced from the first to the second administration. The highest mean score at baseline was for the Emotional Status domain, at 63.45, which dropped to 52.00 after 4 weeks. There were also correlations between changes in CCVUQ-Br scores and pain VAS ratings and CEAP class. With regard to the effect size, total CCVUQ-Br score and ulcer size exhibited high sensitivity, while pain VAS and the majority of the CCVUQ-Br domains had moderate sensitivity.

**Conclusions:**

The CCVUQ-Br questionnaire is sensitive in the Brazilian population and exhibited response to change in the sample tested.

## INTRODUCTION

The importance of studies of the quality of life (QoL) of patients with venous ulcers lies in the fact that chronic ulcers are considered a global epidemic,^1^ affecting around 1% of the adult population.^2^ Venous ulcers of the lower limbs account for around 70% to 90% of chronic ulcer cases.^3^

In Brazil, venous ulcers are considered a serious public health problem, contributing to increased spending by the Brazilian National Health Service (SUS - Sistema Único de Saúde), because of the symptoms and personal limitations, and compromising the QoL of patients.^4^

The Charing Cross Venous Ulcer Questionnaire (CCVUQ) is a disease-specific questionnaire for assessment of QoL in people with venous ulcers that has been considered excellent and promising.^5^^,^ 6 It is recommended for use in a range of scenarios, with the objective of supporting effective measures and assessments of treatment, health promotion, and venous ulcer prevention.^7^ It was created in the United Kingdom and its translation, cultural adaptation, and tests of psychometric properties have been evaluated, generating Chinese, Spanish, and Brazilian versions.^5^^,^ 6 ^,^ 8 ^-^ 11

The Brazilian version is the CCVUQ-Br, comprising 21 items that identify four important health domains: social function, domestic activities, cosmesis, and emotional status. It has elevated internal consistency in terms of correlation of items with the total questionnaire score, good reproducibility because of consistent responses from stable patients, and reasonable to good construct validity, when compared with SF-36 domains.^6^^,^ 11

Notwithstanding the importance of all of the psychometric properties of the CCVUQ-Br that have already been tested, if the intention is to assess changes in QoL over time, another property that must be evaluated is responsiveness, which is a crucial element in assessment of the instrument’s validity.^12^

Responsiveness constitutes testing an instrument’s capacity to measure clinically relevant changes in response to a therapeutic intervention,^12^^,^ 13 which can be accomplished by calculating effect size (ES), indicative of responsiveness or internal sensitivity, and by testing for correlation with other assessment scales, showing responsiveness or external sensitivity.^12^^,^ 14

Internal responsiveness, measured by the ES, evaluates the difference in changes between groups or changes within a single group and may be based on comparisons before and after treatment.^15^

External responsiveness, which is also known as the concurrent validity of an instrument, can be assessed by means of correlations between measures of QoL and other measures or phenomena with clinical relevance, such as an external event, a scale, or a condition, for example.^16^

The relevance of the CCVUQ-Br, the need to validate its responsiveness, and the importance of QoL and the problems caused by venous ulcers are the justifications for conducting this study, the objective of which is to investigate the responsiveness of the CCVUQ- Br venous ulcer QoL questionnaire.

## METHOD

This research project was granted Ethics Committee approval. The voluntary nature of patients’ participation was documented using free and informed consent forms.

The sample comprised people with open venous ulcers due to undergo medical treatment, recruited at venous ulcer care centers over a 1-year period. The Kolmogorov-Smirnov and Shapiro-Wilk tests were used to determine normality of the samples.

No sample size calculation was performed, but values recommended in previous studies were used.^13^ The sample selection technique was non-probabilistic and the final sample comprised 51 patients. Candidates were excluded if they were less than 18 years old; had arterial or lymphatic disorders, psychiatric disorders or dementia; were unable to speak or understand Portuguese; had venous thrombosis, erysipelas, non-venous ulcers, lymphangitis, or diabetes; or were over the age of 60 with cognitive impairment.

The instruments employed in the study were: the CCVUQ-Br,^6^^,^ 11 which assesses QoL in venous ulcer patients on a scale from 0 (zero), for best QoL, to 100 (one hundred), for worst QoL; a pain visual analog scale (pain VAS),^17^ which represents reported pain on a scale from 0 (zero) to 10 (ten), where higher values indicate worse pain; the Global Assessment of Change Scale,^15^ which indicates the patients’ perceptions of the status of their wound on a scale from -5 to 5, with the initial baseline condition set to 0 (zero), so that minus values indicate perceived worsening of the injury; the Clinical, Etiologic, Anatomic, and Pathophysiologic (CEAP)^18^ classification of chronic venous disease, which, in this study, varied from 5 to 6, where 5 indicates a healed venous ulcer and 6 indicates an open venous ulcer.

Patients who fulfilled the inclusion criteria were invited to take part in the study while they waited for their consultations at the healthcare institutions. Presence of venous ulcer was diagnosed by a vascular surgeon. After providing information and signing the free and informed consent form, patients began their participation, which consisted of two contacts with the researchers: at baseline and at study end (4 weeks after starting treatment).

At baseline, the size of the ulcer was measured and participants were given a file containing: a) the CCVUQ-Br; b) a data collection form containing questions on sex, age, time since onset of venous ulcer, current occupation, and educational level; and c) the pain VAS.

After this first contact, participants embarked on the treatment plan prescribed by an angiologist or vascular surgeon. Treatment was chosen by the physician and was administered according to routine practice; frequency and duration of treatment were defined on the basis of medical criteria and are not assessed in this study.

The final analysis was conducted 4 weeks after starting treatment, when the patient attended a return consultation and the physician assessed their CEAP classification once more. At this meeting the participants were requested by a trained researcher to answer the CCVUQ-Br and the pain VAS again and the Global Assessment of Change Scale was administered once more. The size of the ulcer was measured a second time.

The analytical procedures for the study began with input of all data to Excel, which were later exported to the Statistical Package for the Social Sciences (SPSS), version 22.0.

Sociodemographic and clinical data used to trace the profile of the characteristics of the sample were expressed as absolute and relative frequencies (percentages).

The results used to test the instrument’s responsiveness were the CCVUQ-Br total and domain scores, used to calculate the differences over time between the two administrations (4 weeks - baseline), to calculate ES, and to calculate correlations between changes in total and domain CCVUQ-Br scores and CEAP class, pain VAS, ulcer size, and Global Assessment of Change Scale scores.

Descriptive statistics such as sample size, mean, median, minimum, maximum, and standard deviation (SD) were calculated for age, CCVUQ-Br score, pain VAS, and size of ulcer at baseline and after 4 weeks.

Changes over time (4 weeks - baseline) in CCVUQ-Br score, pain VAS ratings, and ulcer size measurements were analyzed by calculating mean and SD of changes and assessing them with the Wilcoxon z and paired Student *t* tests, to determine whether or not the changes were significant.

For internal sensitivity analysis, ES (SD of change and baseline SD) and partial η^2^ of the CCVUQ-Br score, pain VAS, and ulcer size were calculated, considering a 0.05 significance level.

Effect size is a measure that assesses the difference between means of a single variable measured at two times or in different groups. It can be calculated using Cohen’s equation,^19^ in which the mean for the differences between two assessments (final - baseline) is divided by the SD for the differences; this calculation can be performed using a specific, referenced, statistical program.^20^ Another version of ES used in clinical studies is calculated by dividing the mean of the differences between the two assessment times (final - baseline) by the SD of the variable at baseline.^21^^,^ 22 Effect sizes are used to calculate sample size for future studies.

In the present study, ESs were calculated using the differences over time between means for CCVUQ-Br scores, pain VAS rating, and ulcer size, measured at the two time points (baseline and after 4 weeks), using both methods.

With regard to forms of interpretation, there are studies stating that ES can be used to test sensitivity^15^^,^ 23 according to the following interpretation of ES values: ES < 0.5 indicates low sensitivity to change; 0.5 ≤ ES < 0.8 indicates moderate sensitivity to change; and ES ≥ 0.8 indicates high sensitivity to change.^24^

Another method of evaluating ES is to analyze partial η^2^, which refers to the proportion of total variance explained by the assessment times. For this measure, values are interpreted as follows: partial η^2^ from 0.00 to 0.02 indicates small ES; partial η^2^ from 0.02 to 0.13 indicates moderate ES; and partial η^2^ greater than 0.13 indicates large ES.^19^

For analysis of external sensitivity, Spearman correlation coefficients, Pearson correlation coefficients, and Kendall’s tau b were calculated for correlations between change in CCVUQ-Br scores and other scales (pain VAS, CEAP, and Global Assessment of Change Scale) and ulcer size.

## RESULTS

The sample comprised 51 individuals, with ages ranging from 36 to 90 years (mean = 64.53 years; SD = 13.56). Females predominated (62.7%) and the most common educational level category was primary education completed (31.4%). The majority of patients (45.1%) had had an active venous ulcer for more than 1 year, 29.4% were retired because of age, 27.5% were still working, and 25.5% were retired because of illness (Table 1).

**Table 1:** Characteristics of the sample of patients with open chronic venous ulcers.

**Characteristic**	**n**	**%**
Total	51	100.0
Gender		
Female	32	62.7
Male	19	37.3
Educational level		
Illiterate	1	2.0
Functionally illiterate	3	5.9
Primary education uncompleted	5	9.8
Primary education completed	16	31.4
Secondary education uncompleted	12	23.5
Secondary education completed	4	7.8
Higher education uncompleted	8	15.7
Higher education completed	1	2.0
Did not answer	1	2.0
Occupation		
Domestic activities	6	11.8
Unemployed	1	2.0
Employed	14	27.5
Retired due to disease	13	25.5
Retired due to age	15	29.4
Did not know	2	3.9
Time since onset of ulcer		
Less than 2 weeks	3	5.9
From 2 weeks to 1 month	7	13.7
From 1 month to 6 months	14	27.5
From 6 months to 1 year	4	7.8
More than 1 year	23	45.1

The CCVUQ-Br, pain VAS, and ulcer size values were expressed as mean, median, SD, and/or maximum and minimum values at the two assessment times. It was observed that total and domain CCVUQ-Br scores reduced, as did mean ulcer size and pain VAS ratings, which are results that do not indicate clinical deterioration of the sample (Table 2).

**Table 2:** Descriptive summary of the CCVUQ-Br and its domains, the pain VAS, and size of ulcer by time of assessment.

**Scale**	**Time**	**n**	**Mean**	**SD**	**Minimum**	**Maximum**	**Median**
Total	Baseline	51	52.37	15.60	23	85	53
CCVUQ-Br	4 weeks	51	39.71	16.12	19	81	37
CCVUQ-Br	Baseline	51	47.94	20.57	18	86	47
Social Role	4 weeks	51	34.69	18.44	18	86	27
CCVUQ-Br	Baseline	51	52.33	25.26	17	84	55
Domestic Activities	4 weeks	51	39.31	24.75	17	84	25
CCVUQ-Br	Baseline	51	54.20	20.66	21	100	51
Cosmesis	4 weeks	51	41.94	19.94	21	100	41
CCVUQ-Br	Baseline	51	63.45	24.84	21	100	60
Emotional Status	4 weeks	51	52.00	25.52	21	97	45
Pain VAS	Baseline	51	4.08	3.19	0	10	5
	4 weeks	51	2.29	3.00	0	10	1
Size of	Baseline	51	5.14	4.74	0.7	23.12	3
ulcer (cm)	4 weeks	51	2.81	4.12	0	20	1.5

CCVUQ-Br: Brazilian version of the Charing Cross Venous Ulcer Questionnaire; SD: standard deviation; VAS: visual analog scale.

The changes observed were statistically significant for total and domain CCVUQ-Br scores, pain VAS rating, and ulcer size, as shown in Table 3.

**Table 3:** Comparative analysis of the Wilcoxon z and paired Student’s *t* test to detect differences over time in CCVUQ-Br scores, pain VAS ratings, and ulcer size measurements.

**Change on scale** **(4 weeks - baseline)**	**n**	**Wilcoxon *z***	**p**	**Mean change (4 weeks - baseline)**	**SD of change**	**95%CI of change**	**t**	**p**	**Power observed (%)** **at α = 0.05**
Total CCVUQ-Br	51	-4.82	< 0.001	-12.67	14.73	-16.81 to -8.52	-6.14	< 0.001	99.99
CCVUQ-Br Social Role	51	-4.22	< 0.001	-13.25	20.38	-18.99 to -7.52	-4.65	< 0.001	99.53
CCVUQ-Br Domestic Activities	51	-2.85	0.004	-13.02	30.12	-21.49 to -4.55	-3.09	0.003	85.69
CCVUQ-Br Cosmesis	51	-4.22	< 0.001	-12.25	17.44	-17.16 to -7.34	-5.02	< 0.001	99.85
CCVUQ-Br Emotional Status	51	-3.30	0.001	-11.45	22.03	-17.65 to -5.25	-3.71	0.001	95.35
Pain VAS	51	-3.84	< 0.001	-1.78	2.90	-2.60 to -0.97	-4.39	< 0.001	99.06
Ulcer size (cm)	51	-5.58	< 0.001	-2.33	2.90	-3.15 to -1.51	-5.73	< 0.001	99.99

CCVUQ-Br: Brazilian version of the Charing Cross Venous Ulcer Questionnaire; SD: standard deviation; VAS: visual analog scale; 95%CI: 95% confidence interval.

According to the statistical references adopted for the study, the results for ES calculated to determine internal sensitivity show that total CCVUQ-Br score and ulcer size exhibited high sensitivity to change; pain VAS and the Social Role, Cosmesis, and Emotional Status domains of the CCVUQ-Br exhibited moderate sensitivity to change; and the Domestic Activities domain of the CCVUQ-Br exhibited low sensitivity to change. The partial η^2^ analysis returned values varying from 0.160 to 0.430, indicating a large ES, 4 weeks after the start of intervention. These data are shown in Table 4.

**Table 4:** Effect sizes after 4 weeks for CCVUQ-Br, pain VAS, and ulcer size.

**Scale**	**Mean change (4 weeks - baseline)**	**SD of change**	**SD** **baseline**	**Effect size** **(SD of change)**	**95%CI** **Effect size (SD of change)**	**Effect size** **(SD baseline)**	**partial η^2^**
Total CCVUQ-Br	-12.67	14.73	15.60	-0.860	-1.178 to -0.535	-0.812	0.430
CCVUQ-Br Social Role	-13.25	20.38	20.57	-0.651	-0.950 to -0.345	-0.644	0.302
CCVUQ-Br Domestic Activities	-13.02	30.12	25.26	-0.432	-0.717 to -0.143	-0.515	0.160
CCVUQ-Br Cosmesis	-12.25	17.44	20.66	-0.703	-1.007 to -0.393	-0.593	0.335
CCVUQ-Br Emotional Status	-11.45	22.03	24.84	-0.520	-0.810 to -0.225	-0.461	0.216
Pain VAS	-1.78	2.90	3.19	-0.615	-0.912 to -0.313	-0.558	0.279
Ulcer size (cm)	-2.33	2.90	4.74	-0.802	-1.115 to -0.483	-0.492	0.396

CCVUQ-Br: Brazilian version of the Charing Cross Venous Ulcer Questionnaire; SD: standard deviation; VAS: visual analog scale; 95%CI: 95% confidence interval.

With regard to the CEAP analysis, it is important to consider that all 51 participants in the initial sample were classified as having venous disease at CEAP class 6. After 4 weeks, 43.1% (95% confidence interval [95%CI] 30.2%-56.8%, n = 22) had reduced to CEAP 5, and 56.9% (95%CI 43.2%-69.8%, n = 29) were still at CEAP 6.

The results for the Global Assessment of Change Scale show that the majority of patients (94.2%) reported an improvement in their condition.

Table 5 contains results for the tests of external sensitivity, listing correlations between the instrument (CCVUQ-Br and its domains), the scales (pain VAS, Global Assessment of Change Scale, and CEAP), and clinical measurements (ulcer size).

**Table 5:** Correlations between changes in CCVUQ-Br total and domain scores and changes in pain VAS rating, Global Assessment of Change Scale score, ulcer size measurements, and CEAP class.

**Change in**	**Change in**	**n**	**Pearson**		**Spearman**	
			**Correlation**	**p**	**Correlation**	**p**
Total CCVUQ-Br	Pain VAS	51	0.498	< 0.001	0.475	< 0.001
Total CCVUQ-Br	Ulcer size (cm)	51	0.065	0.652	0.173	0.225
Total CCVUQ-Br	Global Assessment of Change Scale	51	-0.290	0.039	-0.232	0.102
Total CCVUQ-Br	CEAP	51	0.427	0.002	0.416	0.002
CCVUQ-Br Social Role	Pain VAS	51	0.363	0.009	0.342	0.014
CCVUQ-Br Social Role	Ulcer size (cm)	51	-0.075	0.603	0.115	0.421
CCVUQ-Br Social Role	Global Assessment of Change Scale	51	-0.309	0.028	-0.201	0.157
CCVUQ-Br Social Role	CEAP	51	0.425	0.002	0.445	0.001
CCVUQ-Br Domestic Activities	Pain VAS	51	0.384	0.005	0.372	0.007
CCVUQ-Br Domestic Activities	Ulcer size (cm)	51	0.254	0.072	0.389	0.005
CCVUQ-Br Domestic Activities	Global Assessment of Change Scale	51	-0.153	0.283	-0.082	0.569
CCVUQ-Br Domestic Activities	CEAP	51	0.375	0.007	0.350	0.012
CCVUQ-Br Cosmesis	Pain VAS	51	0.392	0.004	0.438	0.001
CCVUQ-Br Cosmesis	Ulcer size (cm)	51	-0.050	0.726	-0.053	0.711
CCVUQ-Br Cosmesis	Global Assessment of Change Scale	51	-0.109	0.447	-0.049	0.731
CCVUQ-Br Cosmesis	CEAP	51	0.166	0.245	0.163	0.254
CCVUQ-Br Emotional Status	Pain VAS	51	0.166	0.245	0.131	0.360
CCVUQ-Br Emotional Status	Ulcer size (cm)	51	0.009	0.950	-0.073	0.612
CCVUQ-Br Emotional Status	Global Assessment of Change Scale	51	-0.135	0.344	-0.147	0.302
CCVUQ-Br Emotional Status	CEAP	51	0.149	0.297	0.137	0.337

CCVUQ-Br: Brazilian version of the Charing Cross Venous Ulcer Questionnaire; CEAP: Clinical, Etiologic, Anatomic, and Pathophysiologic classification; VAS: visual analog scale.

In a first analysis, it was observed that the total CCVUQ-Br score had reasonable positive correlations with pain VAS and CEAP classification, no correlation with ulcer size, and a weak correlation with the Global Assessment of Change Scale.

With regard to correlations with the CCVUQ-Br domains, there was a tendency to replicate what was observed with the CCVUQ-Br score; notable exceptions were a correlation between Domestic Activities and ulcer size, an absence of correlation between Cosmesis and CEAP classification, and no correlation between Emotional Status and pain VAS or CEAP classification.

Internal consistency was analyzed for both times of administration of the CCVUQ-Br, both with and without weighting, by calculating Cronbach’s α values. Values of α > 0.660 were observed and values for total CCVUQ-Br score and for the domains increased in the comparison between baseline and 4 weeks after starting treatment.

## DISCUSSION

It is believed that since venous ulcer is a chronic disease, the outcomes should not be assessed exclusively with traditional epidemiological measures, emphasizing the importance of testing the sensitivity of the instruments used.^25^

The CCVUQ-Br has already been translated to Portuguese and cross-culturally adapted for Brazil^6^ and its reliability and validity have been tested^11^; but its sensitivity still needs to be verified.

### Discussion of sensitivity methods

There is no consensus in the literature on how to investigate the sensitivity of assessment instruments.^26^ However, it is agreed that for a measure to be sensitive, it must exhibit consistent change over time or be compared with another measure of known value.^27^ This study assessed the CCVUQ-Br in relation to change over time and these changes were correlated with changes measured by other scales.

A sensitivity assessment method may include analysis of changes before and after treatment,^1^ which is the model adopted for the present study with the CCVUQ-Br questionnaire. In this case, the time elapsed between the first and second administrations of the questionnaire was 4 weeks. However, this interval is not defined methodologically, varying from 2 weeks to 1 year in some studies.^28^ We chose 4 weeks because we considered that this period is sufficient for patients undergoing a therapeutic intervention to exhibit clinical improvement, with a reduction in ulcer size.^29^

When comparing measures of health-related quality of life (HRQoL) with other clinically relevant measures, one option is comparison with the Global Assessment of Change Scale, offering the principal advantage of comparison with a measure of change that is based on the patient’s own perspective.^15^ Use of the Global Assessment of Change Scale agreed with the present study, although in this case other clinically relevant measures were also used, such as the pain VAS, measurement of ulcer size, and changes in CEAP classification.

We used the pain VAS because we consider that pain is a common characteristic in patients with venous ulcers and many patients describe it as the symptom that has the greatest impact on their QoL.^30^

Along the same lines, the comparison with change in ulcer size was chosen because this parameter is evidence of clinical improvement or deterioration. In turn, comparison with change in CEAP classification was included because this classification is considered an indication of the severity of chronic venous disease and also of HRQoL.^31^

Since there is no methodological definition for calculating sample size when validating sensitivity, the recommendation that studies of psychometric properties should not have samples of less than 50 individuals was followed.^13^ A suggestion made in another study,^25^ recommending that when analyzing questionnaires a minimum of 10 individuals should be assessed per domain, was also followed.

### Discussion of the statistical method

Still with relation to sensitivity, one study distinguished between two principal types of capacity to respond to change: internal, determined by analysis of ES, and external, determined in terms of correlations with other scales.^13^ In the present study, the CCVUQ-Br was assessed using tests of ES and correlations with other scales, in order to conduct a more comprehensive analysis of sensitivity.

The ES method is considered the most appropriate for testing an instrument’s sensitivity, since, in addition to the method’s simplicity, it also provides references for specific and generic instruments that can improve interpretation of therapeutic changes and changes related to health status.^15^

Statistical analysis by means of testing ES is described in a study^32^ that attempted to determine whether the instrument under investigation was sensitive to change in variables after an intervention. With the same objective, correlations between changes in variables were also conducted.

The study design referred to above is similar to that employed in the present study and indicates that recommendations were followed with regard to using the ES statistics to analyze changes in CCVUQ-Br scores. However, we also used the paired Student *t* and Wilcoxon *z* tests to analyze the same variable. This choice does not contradict the earlier recommendations, since, in other studies the *t* test has been cited as the most widely used statistical method for calculating sensitivity, in conjunction with Wilcoxon’s *z*.^15^

### Discussion of the results

The characteristics of the sample studied are in line with earlier studies with venous ulcer patients, including studies using the CCVUQ.^5^^,^ 8

There are also similarities in terms of the ulcer patients’ occupations, the majority of whom were retired in the present study (54.9%), with a large proportion retired because of the disease (25.5%).^33^

Observing reductions in the pain VAS rating and ulcer size measurements, it was also observed that there were improvements in patients’ QoL, as indicated by reductions in CCVUQ-Br scores. Improved QoL after treatment was also observed in another study, in which QoL improved after 8 months’ treatment with Unna boots.^34^

### Discussion of responsiveness results

Effect sizes and partial η^2^ were calculated to analyze the significance of the changes observed in total and domain CCVUQ-Br scores, in pain VAS rating, and in ulcer size measurements. The results ranged from ES with low sensitivity for one of the questionnaire domains to moderate and high sensitivity for the other domains, for pain VAS, and for ulcer size. This type of variation in ES values, even between different domains of the same questionnaire, was also observed in a study about an instrument to assess wounds.^15^

Analysis of the results for correlations between changes in CCVUQ-Br scores and changes in other clinically relevant measures showed that the highest correlations were between the pain VAS rating and CEAP classification. Lower correlations or absence of correlation were observed with change in ulcer size and the Global Assessment of Change Scale, which might be explained by a study that compared QoL in patients with healed ulcers and active ulcers and concluded that healing of the ulcer did not contribute to improving patients’ QoL.^35^ The low correlation between these two measures is possibly because a more significant change in terms of the characteristics of lesions is necessary.

## CONCLUSIONS

The CCVUQ-Br questionnaire on QoL in venous ulcer patients is sensitive for longitudinal analysis when used in a Brazilian population.
