# Supplementary material for: Responsiveness of the CCVUQ-Br quality of life questionnaire in chronic venous ulcer patients
Source: J Vasc Bras. 2020 Jan 9;19:e20190047. doi: 10.1590/1677-5449.190047 (PMC6956623; doi:10.1590/1677-5449.190047)
Supplement: Translation

# Translation: Responsividade do questionário de qualidade de vida CCVUQ-Br em portadores de úlcera venosa crônica

**Authors:** Renata Cardoso Couto, Flávia de Jesus Leal, Guilherme Benjamin Brandão Pitta, Solange Andreoni

**Affiliations:** 1 Universidade Federal de São Paulo – UNIFESP, São Paulo, SP, Brasil.; 2 Universidade Estadual de Ciências da Saúde de Alagoas – UNCISAL, Maceió, AL, Brasil.

**Keywords:** questionário de úlcera venosa, sensibilidade e especificidade, qualidade de vida

## Abstract

**Contexto:**

A responsividade consiste na capacidade de um instrumento em verificar se a pontuação reflete a variabilidade ocorrida na vida do paciente decorrente de uma intervenção. O CCVUQ-Br foi validado na língua portuguesa e necessita ter sua responsividade verificada. Quando finalizado este estudo, o CCVUQ-Br será utilizado como um instrumento capaz de perceber e refletir, na sua pontuação, as mudanças ocorridas na qualidade de vida do portador de úlcera venosa.

**Objetivos:**

Avaliar a responsividade do CCVUQ-Br.

**Métodos:**

Estudo de intervenção longitudinal, realizado em centros públicos e privados para pacientes com úlcera venosa. A amostra foi composta por portadores de úlcera venosa crônica submetidos à conduta terapêutica, tendo como variáveis as pontuações do CCVUQ-Br e de seus domínios, da escala visual analógica da dor (EVA dor) e da Escala de Avaliação Global de Mudança, além da classificação CEAP e o tamanho da úlcera. O CCVUQ-Br foi aplicado em 51 indivíduos submetidos a conduta terapêutica, recrutados de forma aleatória. Após 4 semanas, o CCVUQ-Br foi reaplicado.

**Resultados:**

Houve diminuição das pontuações médias do CCVUQ-Br entre os dois momentos de aplicação, sendo que, no momento basal, a maior média de pontuação foi a do domínio Estado Emocional, com 63,45, diminuindo, após 4 semanas, para 52,00. Ainda apresentou correlações das mudanças com EVA dor e CEAP. Em relação ao tamanho do efeito, pode-se considerar que pontuação total do CCVUQ-Br e tamanho da úlcera apresentaram sensibilidade elevada, enquanto EVA dor e a maioria dos domínios do CCVUQ-Br apresentaram sensibilidade moderada .

**Conclusões:**

O questionário CCVUQ-Br é sensível na população brasileira, apresentando garantia de resposta à amostra testada.

## INTRODUÇÃO

Estudos sobre qualidade de vida (QV) em pacientes com úlcera venosa têm sua importância, por ser a úlcera crônica considerada uma epidemia mundial^1^, que atinge cerca de 1% da população adulta^2^; entre as úlceras crônicas, a úlcera venosa de membros inferiores representa cerca de 70% a 90% dos casos^3^.

No Brasil, a úlcera venosa é considerada um sério problema de saúde pública, contribuindo para aumentar os gastos no Sistema Único de Saúde (SUS), pelos sintomas e limitações pessoais, levando ao comprometimento da QV do portador^4^.

O instrumento Charing Cross Venous Ulcer Questionnaire (CCVUQ) é um questionário específico de avaliação da QV em indivíduos com úlcera venosa considerado excelente e promissor^5^^,^ 6, sendo recomendado para uso em diferentes cenários, com o objetivo de subsidiar efetivas avaliações e medidas de tratamento, promoção de saúde e prevenção da úlcera venosa^7^. Foi criado no Reino Unido e teve sua tradução, adaptação cultural e testes das propriedades psicométricas avaliados, gerando as versões chinesa, espanhola e brasileira^5^^,^ 6 ^,^ 8 ^-^ 11.

O CCVUQ-Br é a versão brasileira, sendo composto por 21 itens que identificam quatro domínios importantes da saúde: interação social, atividades domésticas, estética e estado emocional, apresentando consistência interna elevada, devido a correlação dos itens com a pontuação total do questionário; alta reprodutibilidade, devido a consistência das respostas em indivíduos estáveis; e validade de constructo de razoável a boa quando comparado com os domínios do SF 36^6^^,^ 11.

Apesar da importância de todas as propriedades psicométricas do CCVUQ-Br já testadas, quando se deseja avaliar as mudanças na QV ao longo do tempo, é necessário que mais uma seja analisada, a responsividade, pois se trata de um elemento crucial para avaliar a validade do instrumento^12^.

Responsividade consiste em testar a capacidade de um instrumento de medir mudanças clinicamente importantes em resposta a uma intervenção terapêutica^12^^,^ 13, podendo ser obtida pela verificação do tamanho do efeito (TE) (responsividade ou sensibilidade interna) e pela correlação com outras avaliações ou escalas (responsividade ou sensibilidade externa)^12^^,^ 14.

A responsividade interna, medida pelo TE, avalia a diferença da mudança entre grupos ou mudanças dentro de um grupo, podendo incluir comparações antes ou após tratamento^15^.

A responsividade externa, conhecida também como validação concorrente do instrumento, pode ser avaliada por meio de correlações entre as medidas de QV e outras medidas ou fenômenos de relevância clínica, por exemplo, um evento externo, uma escala ou uma condição^16^.

Diante da relevância do CCVUQ-Br, da necessidade de verificação da responsividade desse instrumento, e da importância da QV e dos problemas gerados pela úlcera venosa, justificou-se este estudo, que teve como objetivo investigar a responsividade do questionário de QV na úlcera venosa, o CCVUQ- Br.

## MÉTODO

Este projeto de pesquisa foi aprovado pelo Comitê de Ética. A participação voluntária dos indivíduos foi documentada em termo de consentimento livre e esclarecido (TCLE).

A amostra foi formada por indivíduos portadores de úlcera venosa aberta a serem submetidos a tratamento médico e foi recrutada em centros de assistência a portadores de úlcera venosa no período de 1 ano. Para definir a normalidade das amostras, foram utilizados os testes de Kolmogorov-Smirnov e Shapiro-Wilk.

Não houve cálculo do tamanho da amostra, seguindo-se valores recomendados por estudos anteriores^13^. A técnica de amostragem foi não probabilística, e a amostra final de 51 indivíduos. Foram excluídos indivíduos menores de 18 anos; com alterações arteriais e linfáticas associadas, distúrbio psiquiátrico ou quadro demencial; que não falavam ou compreendiam a língua portuguesa; portadores de trombose venosa, erisipela, úlceras não venosas, linfangite, diabetes; e indivíduos com idade igual ou superior a 60 anos que tivessem alteração cognitiva.

Os instrumentos utilizados no estudo foram: CCVUQ-Br^6^^,^ 11, o qual avalia QV na úlcera venosa e varia de 0 (zero), para melhor QV, a 100 (cem), para pior QV; escala visual analógica da dor (EVA dor)^17^, que indica a dor referida, com variação de 0 (zero) a 10 (dez), na qual valores crescentes indicam piora da dor; Escala de Avaliação Global de Mudança^15^, que indica a percepção do indivíduo em relação à sua ferida e varia de -5 a 5, sendo a referência inicial 0 (zero), com valores menores indicando percepção de piora da ferida; classificação Clinical, Etiologic, Anatomic, Pathophysiologic (CEAP)^18^, baseada na clínica, etiologia, anatomia e fisiopatologia da doença venosa crônica, podendo variar, no estudo, entre 5 e 6, sendo a classificação 5 referente a úlcera venosa cicatrizada, e a classificação 6 a úlcera venosa aberta.

Os indivíduos que atenderam aos critérios de inclusão foram convidados a participar do estudo no momento em que aguardavam a consulta nas instituições de saúde. A presença da úlcera venosa era diagnosticada pelo cirurgião vascular. Após a prestação de informações e a assinatura do TCLE, os indivíduos iniciavam sua participação, que era executada em dois contatos da amostra com os pesquisadores: momento basal e momento final (após 4 semanas do início da conduta terapêutica).

No momento basal, era mensurado o tamanho da úlcera e os indivíduos recebiam do pesquisador uma pasta contendo: a) o CCVUQ-Br; b) um formulário de coleta de dados contendo perguntas referentes a sexo, idade, tempo de úlcera venosa, ocupação atual e grau de escolaridade; e c) a EVA dor.

Após o contato, os indivíduos iniciavam a conduta terapêutica prescrita por angiologista ou cirurgião vascular. A terapêutica era de escolha médica e foi rotineiramente aplicada; frequência e tempo de conduta terapêutica foram definidos por critérios médicos e não foram avaliados na presente pesquisa.

O momento de análise final ocorreu 4 semanas após o início da conduta terapêutica, no retorno à consulta médica, quando o médico voltava a verificar a classificação CEAP. Nesse momento, os sujeitos foram orientados por um pesquisador treinado a responder novamente o CCVUQ-Br e a EVA dor, além de preencherem a Escala de Avaliação Global de Mudança. Houve a mensuração do tamanho da úlcera pela segunda vez.

Na realização dos procedimentos analíticos do estudo, todos os dados foram inicialmente armazenados no programa Excel e posteriormente transferidos para o software Statistical Package for the Social Sciences (SPSS), versão 22.0.

Os dados sócio demográficos e clínicos utilizados na caracterização da amostra foram descritos em frequências absolutas e relativas (porcentagens).

Os resultados na verificação da responsividade foram obtidos por meio das pontuações total e por domínios do CCVUQ-Br, usadas para o cálculo das diferenças no tempo entre as duas aplicações (4 semanas - basal), para o cálculo do TE, e para o cálculo da correlação das mudanças no CCVUQ-Br e em seus domínios entre si, com a CEAP, com a EVA dor, com o tamanho da úlcera e com a escala de avaliação global de mudança.

Utilizaram-se estatísticas descritivas, incluindo tamanho da amostra, média, mediana, mínimo e máximo e desvio padrão (DP), na descrição de idade, pontuações do CCVUQ-Br, EVA dor e tamanho da úlcera, no momento basal e após 4 semanas,

Para avaliar as mudanças no tempo (4 semanas - basal) das pontuações do CCVUQ-Br, EVA dor e tamanho da úlcera, foram calculados média e DP das mudanças, e aplicaram-se os testes *z* de Wilcoxon e *t* de Student pareado, a fim de se verificar se as mudanças foram significativas ou não.

Para análise da sensibilidade interna, foram descritos valores de TE (DP da mudança e DP basal) e η^2^ parcial das pontuações do CCVUQ-Br, EVA dor e tamanho da úlcera, considerando-se nível de significância de 0,05.

O TE é a medida que avalia a mudança entre as médias de uma mesma variável em dois momentos de avaliações diferentes ou em grupos diferentes. Pode ser calculado pela equação de Cohen^19^, em que a média das diferenças entre dois momentos de avaliação (final - basal) é dividida pelo DP das diferenças; esse cálculo pode ser executado por meio do programa estatístico, específico e referenciado^20^. Outro TE utilizado em estudos clínicos é calculado dividindo-se a média das diferenças entre dois momentos de avaliação (final - basal) pelo DP da variável no momento basal^21^^,^ 22. TEs são utilizados para se calcular tamanho de amostra em futuros estudos.

No presente estudo, o TE foi calculado considerando-se as diferenças das médias das pontuações do CCVUQ-Br, da EVA dor e do tamanho da úlcera, verificadas ao longo do tempo, entre dois momentos de avaliação (basal e após 4 semanas), pelos dois métodos.

Entre as formas de interpretação, há estudos que sugerem que o TE seja utilizado na verificação da sensibilidade^15^^,^ 23, de acordo com a seguinte interpretação dos valores de TE: TE < 0,5 significa sensibilidade baixa à mudança; 0,5 ≤ TE < 0,8 significa sensibilidade moderada à mudança; e TE ≥ 0,8 significa sensibilidade alta à mudança^24^.

Outra forma de avaliar o TE é a análise do η^2^ parcial, que se refere à proporção da variância total explicada pelos momentos de avaliação. Os valores utilizados na análise dessa medida são: η^2^ parcial entre 0,00 e 0,02 significa TE pequeno; η^2^ parcial entre 0,02 e 0,13 significa TE moderado; e η^2^ parcial maior que 0,13 significa TE grande^19^

Para análise da sensibilidade externa, foram descritas as correlações da mudança entre o CCVUQ-Br, escalas (EVA dor, CEAP e Escala de Avaliação Global de Mudança) e tamanho da úlcera, verificadas pelos coeficientes de correlação de Spearman, Pearson e tau b Kendall.

## RESULTADOS

A amostra foi composta por 51 indivíduos, com idade variando de 36 a 90 anos (média = 64,53 anos; DP = 13,56). Houve predominância do gênero feminino (62,7%) e do ensino fundamental completo (31,4%) como grau de escolaridade. A maioria dos pacientes (45,1%) apresentou úlcera venosa ativa por mais de 1 ano, 29,4% encontravam-se aposentados por idade, 27,5% mantinham suas atividades laborativas, e 25,5% estavam aposentados pela doença (Tabela 1).

**Tabela 1:** Características dos portadores de úlcera venosa crônica aberta avaliados.

**Característica**	**n**	**%**
Total	51	100,0
Gênero		
Feminino	32	62,7
Masculino	19	37,3
Escolaridade		
Analfabeto	1	2,0
Analfabeto funcional	3	5,9
Fundamental incompleto	5	9,8
Fundamental completo	16	31,4
Ensino médio incompleto	12	23,5
Ensino médio completo	4	7,8
Ensino superior incompleto	8	15,7
Ensino superior completo	1	2,0
Não respondeu	1	2,0
Ocupação		
Atividades domésticas	6	11,8
Desempregado	1	2,0
Empregado	14	27,5
Aposentado pela doença	13	25,5
Aposentado por idade	15	29,4
Não sabe	2	3,9
Tempo de úlcera		
Inferior a 2 semanas	3	5,9
De 2 semanas a 1 mês	7	13,7
De 1 a 6 meses	14	27,5
De 6 meses a 1 ano	4	7,8
Mais que 1 ano	23	45,1

Valores de CCVUQ-Br, EVA dor e tamanho da úlcera foram descritos por média, mediana, DP, máximo e mínimo, nos dois momentos de avaliação da amostra, destacando-se a diminuição das médias da pontuação total e dos domínios do CCVUQ-Br, além da diminuição das médias do tamanho da úlcera e da pontuação da EVA dor, resultados estes que não indicam piora clínica da amostra estudada e que estão descritos na Tabela 2.

**Tabela 2:** Resumo descritivo do CCVUQ-Br e de seus domínios, da EVA dor e do tamanho de úlcera segundo os momentos de avaliação.

**Escala**	**Momento**	**n**	**Média**	**DP**	**Mínimo**	**Máximo**	**Mediana**
CCVUQ-Br	Basal	51	52,37	15,60	23	85	53
Total	4 semanas	51	39,71	16,12	19	81	37
CCVUQ-Br	Basal	51	47,94	20,57	18	86	47
Interação Social	4 semanas	51	34,69	18,44	18	86	27
CCVUQ-Br	Basal	51	52,33	25,26	17	84	55
Atividades Domésticas	4 semanas	51	39,31	24,75	17	84	25
CCVUQ-Br	Basal	51	54,20	20,66	21	100	51
Estética	4 semanas	51	41,94	19,94	21	100	41
CCVUQ-Br	Basal	51	63,45	24,84	21	100	60
Estado Emocional	4 semanas	51	52,00	25,52	21	97	45
EVA dor	Basal	51	4,08	3,19	0	10	5
	4 semanas	51	2,29	3,00	0	10	1
Tamanho da	Basal	51	5,14	4,74	0,7	23,12	3
úlcera (cm)	4 semanas	51	2,81	4,12	0	20	1,5

CCVUQ-Br: versão brasileira do Charing Cross Venous Ulcer Questionnaire; DP: desvio padrão; EVA: escala visual analógica.

É relevante considerar que as mudanças analisadas foram estatisticamente significativas para o CCVUQ-Br e seus domínios, EVA dor e tamanho da úlcera, conforme descrito na Tabela 3.

**Tabela 3:** Análise comparativa entre os testes *z* Wilcoxon e *t* de Student pareado para avaliar diferenças no tempo entre CCVUQ-Br, EVA dor e tamanho da úlcera.

**Mudança na escala** **(4 sem - basal)**	**n**	***z* de Wilcoxon**	**p**	**Média da mudança (4 sem - basal)**	**DP da mudança**	**IC95% da mudança**	**t**	**p**	**Poder observado (%)** **com α = 0,05**
CCVUQ-Br Total	51	-4,82	< 0,001	-12,67	14,73	-16,81 a -8,52	-6,14	< 0,001	99,99
CCVUQ-Br Interação Social	51	-4,22	< 0,001	-13,25	20,38	-18,99 a -7,52	-4,65	< 0,001	99,53
CCVUQ-Br Atividades Domésticas	51	-2,85	0,004	-13,02	30,12	-21,49 a -4,55	-3,09	0,003	85,69
CCVUQ-Br Estética	51	-4,22	< 0,001	-12,25	17,44	-17,16 a -7,34	-5,02	< 0,001	99,85
CCVUQ-Br Estado Emocional	51	-3,30	0,001	-11,45	22,03	-17,65 a -5,25	-3,71	0,001	95,35
EVA dor	51	-3,84	< 0,001	-1,78	2,90	-2,60 a -0,97	-4,39	< 0,001	99,06
Tamanho da úlcera (cm)	51	-5,58	< 0,001	-2,33	2,90	-3,15 a -1,51	-5,73	< 0,001	99,99

CCVUQ-Br: versão brasileira do Charing Cross Venous Ulcer Questionnaire; DP: desvio padrão; EVA: escala visual analógica; IC95%: intervalo de confiança de 95%.

No que diz respeito ao TE, na verificação da sensibilidade interna, e considerando-se as referências estatísticas utilizadas no estudo, pode-se considerar que pontuação total do CCVUQ-Br e tamanho da úlcera apresentaram sensibilidade elevada à mudança; EVA dor e os domínios Interação Social, Estética e Estado Emocional do CCVUQ-Br apresentaram sensibilidade moderada à mudança; e o domínio Atividades Domésticas do CCVUQ-Br apresentou baixa sensibilidade à mudança. Na análise pelo η^2^ parcial, encontramos valores variando entre 0,160 e 0,430, significando TE grande, após 4 semanas do início da intervenção. Esses dados estão demonstrados na Tabela 4.

**Tabela 4:** Tamanhos do efeito após 4 semanas para CCVUQ-Br, EVA dor e tamanho da úlcera.

**Escala**	**Média da mudança (4 sem - basal)**	**DP da mudança**	**DP** **basal**	**Tamanho do efeito** **(DP da mudança)**	**IC95%** **Tamanho do efeito (DP da mudança)**	**Tamanho do efeito** **(DP basal)**	**η^2^ parcial**
CCVUQ-Br Total	-12,67	14,73	15,60	-0,860	-1,178 a -0,535	-0,812	0,430
CCVUQ-Br Interação Social	-13,25	20,38	20,57	-0,651	-0,950 a -0,345	-0,644	0,302
CCVUQ-Br Atividades Domésticas	-13,02	30,12	25,26	-0,432	-0,717 a -0,143	-0,515	0,160
CCVUQ-Br Estética	-12,25	17,44	20,66	-0,703	-1,007 a -0,393	-0,593	0,335
CCVUQ-Br Estado Emocional	-11,45	22,03	24,84	-0,520	-0,810 a -0,225	-0,461	0,216
EVA dor	-1,78	2,90	3,19	-0,615	-0,912 a -0,313	-0,558	0,279
Tamanho da úlcera (cm)	-2,33	2,90	4,74	-0,802	-1,115 a -0,483	-0,492	0,396

CCVUQ-Br: versão brasileira do Charing Cross Venous Ulcer Questionnaire; DP: desvio padrão; EVA: escala visual analógica; IC95%: intervalo de confiança de 95%.

Na análise do CEAP, considerando-se a amostra inicial de 51 indivíduos, é relevante considerar que, no início, todos os indivíduos eram classificados na doença venosa com CEAP 6. Após 4 semanas, 43,1% (intervalo de confiança de 95% [IC95%] 30,2%-56,8%, n = 22) diminuíram para CEAP 5, e 56,9% (IC95% 43,2%-69,8%, n = 29) permaneceram no CEAP 6.

Quanto aos resultados da Escala de Avaliação Global de Mudança, os resultados evidenciaram que a maioria refere uma condição de melhora, relatada por 94,2% da amostra.

A Tabela 5, usada na verificação da sensibilidade externa, descreve as correlações existentes entre o instrumento (CCVUQ-Br e seus domínios), as escalas (EVA dor, Escala de Avaliação Global de Mudança e CEAP) e a mensuração (tamanho da úlcera).

**Tabela 5:** Correlações das mudanças no CCVUQ-Br total e em seus domínios com aquelas ocorridas em EVA dor, Escala de Avaliação Global de Mudança, tamanho da úlcera, e CEAP.

**Mudança em**	**Mudança em**	**n**	**Pearson**		**Spearman**	
			**Correlação**	**p**	**Correlação**	**p**
CCVUQ-Br Total	EVA dor	51	0,498	< 0,001	0,475	< 0,001
CCVUQ-Br Total	Tamanho da úlcera (cm)	51	0,065	0,652	0,173	0,225
CCVUQ-Br Total	Escala Global de Mudança	51	-0,290	0,039	-0,232	0,102
CCVUQ-Br Total	CEAP	51	0,427	0,002	0,416	0,002
CCVUQ-Br Interação Social	EVA dor	51	0,363	0,009	0,342	0,014
CCVUQ-Br Interação Social	Tamanho da úlcera (cm)	51	-0,075	0,603	0,115	0,421
CCVUQ-Br Interação Social	Escala de Avaliação Global de Mudança	51	-0,309	0,028	-0,201	0,157
CCVUQ-Br Interação Social	CEAP	51	0,425	0,002	0,445	0,001
CCVUQ-Br Atividades Domésticas	EVA dor	51	0,384	0,005	0,372	0,007
CCVUQ-Br Atividades Domésticas	Tamanho da úlcera (cm)	51	0,254	0,072	0,389	0,005
CCVUQ-Br Atividades Domésticas	Escala de Avaliação Global de Mudança	51	-0,153	0,283	-0,082	0,569
CCVUQ-Br Atividades Domésticas	CEAP	51	0,375	0,007	0,350	0,012
CCVUQ-Br Estética	EVA dor	51	0,392	0,004	0,438	0,001
CCVUQ-Br Estética	Tamanho da úlcera (cm)	51	-0,050	0,726	-0,053	0,711
CCVUQ-Br Estética	Escala de Avaliação Global de Mudança	51	-0,109	0,447	-0,049	0,731
CCVUQ-Br Estética	CEAP	51	0,166	0,245	0,163	0,254
CCVUQ-Br Estado Emocional	EVA dor	51	0,166	0,245	0,131	0,360
CCVUQ-Br Estado Emocional	Tamanho da úlcera (cm)	51	0,009	0,950	-0,073	0,612
CCVUQ-Br Estado Emocional	Escala Global de Mudança	51	-0,135	0,344	-0,147	0,302
CCVUQ-Br Estado Emocional	CEAP	51	0,149	0,297	0,137	0,337

CCVUQ-Br: versão brasileira do Charing Cross Venous Ulcer Questionnaire; CEAP: classificação Clinical, Etiologic, Anatomic, Pathophysiologic; EVA: escala visual analógica.

Em primeira análise, verificou-se que, em relação ao CCVUQ-Br, foram encontradas correlações positivas e razoáveis com a EVA dor e a classificação CEAP, ausência de correlação com o tamanho da úlcera, e correlação fraca com a Escala de Avaliação Global de Mudança.

Em relação às correlações com os domínios do CCVUQ-Br, houve uma tendência a repetição do que ocorreu nas correlações com a pontuação total do CCVUQ-Br; exceções podem ser notadas com a presença de correlação entre o domínio Atividades Domésticas e o tamanho da úlcera, além da ausência de correlação do domínio Estética com a classificação CEAP e do domínio Estado Emocional com a EVA dor e a classificação CEAP.

A consistência interna foi analisada nos dois momentos de aplicação do CCVUQ-Br, de forma ponderada e não ponderada, por meio da determinação dos valores de α de Cronbach. Foram notados valores de α > 0,660 e aumento dos valores na comparação entre o momento basal e após 4 semanas, tanto na pontuação total do CCVUQ-Br quanto nos seus domínios.

## DISCUSSÃO

Entende-se que, por ser a úlcera venosa uma doença crônica, o desfecho esperado não deve ser avaliado somente por medidas epidemiológicas tradicionais, elevando-se a importância da verificação da sensibilidade dos instrumentos utilizados^25^.

O CCVUQ-Br já foi traduzido e adaptado culturalmente ao português do Brasil^6^, bem como teve sua confiabilidade e validade testadas^11^; no entanto, é necessária a avaliação da sensibilidade.

### Discussão do método de sensibilidade

Não há consenso na literatura sobre como investigar a sensibilidade de um instrumento de avaliação^26^; entretanto, é sabido que, para uma medida ser sensível, deve apresentar mudanças consistentes ao longo do tempo ou ser comparada a outra medida de valor conhecido^27^. No presente estudo, o CCVUQ-Br foi avaliado em relação à sua mudança ao longo do tempo e teve suas mudanças correlacionadas a mudanças em outras escalas.

O método de avaliação da sensibilidade pode incluir a análise das mudanças antes e após o tratamento^1^, modelo seguido no presente estudo com o questionário CCVUQ-Br. Neste caso, o tempo decorrido entre a primeira e a segunda aplicação do questionário foi de 4 semanas; no entanto, esse período não está metodologicamente definido, variando em alguns estudos entre 2 semanas e 1 ano^28^. O tempo de 4 semanas foi utilizado por entendermos que, para pacientes submetidos a intervenção terapêutica, esse intervalo é suficiente para melhora clínica, com redução do tamanho da úlcera^29^.

Ao comparar as medidas de qualidade de vida relacionada à saúde (QVRS) com outras medidas de relevância clínica, pode- se levar em consideração a comparação com a Escala de Avaliação Global de Mudança, tendo como principal vantagem a comparação com uma medida de mudança avaliada de acordo com a perspectiva do indivíduo^15^. O uso da Escala de Avaliação Global de Mudança corrobora com o presente estudo, sendo que neste caso, outras medidas de relevância clínica também foram utilizadas, tais como EVA dor, mensuração do tamanho da úlcera e alterações na classificação CEAP.

A escolha pelo uso da escala EVA dor justifica-se por entendermos que a dor, em pacientes com úlcera venosa, é uma característica frequente, sendo descrita por muitos pacientes como o sintoma de maior impacto em sua QV^30^.

Do mesmo modo, a comparação com a variação no tamanho da úlcera decorre do fato de ser este um parâmetro que evidencia a melhora ou piora clínica do paciente; já a comparação com as alterações na classificação CEAP foi realizada porque essa classificação é considerada um indicativo de severidade da doença venosa crônica e também de QVRS^31^.

Como não há uma definição metodológica para cálculo amostral na verificação da sensibilidade, foi seguida recomendação obtida em estudo das propriedades psicométricas de não ter uma amostra menor que 50 indivíduos^13^. Também foram seguidas sugestões de outro estudo para que fosse considerado, em análise de questionários, o mínimo de 10 indivíduos para cada domínio^25^.

### Discussão do método estatístico

Ainda nesse contexto de considerações em relação à sensibilidade, um estudo distinguiu dois grandes tipos de capacidade de resposta à mudança: interna, obtida pela análise do TE, e a externa, obtida por meio de correlações com outras escalas^13^. No presente estudo com o CCVUQ-Br, os testes de TE e as correlações com outras escalas foram executados buscando uma maior abrangência na análise da sensibilidade.

O método TE é conhecido como o mais apropriado para se testar a sensibilidade de um instrumento, pois, além de ser um método simples, promove referências para instrumentos específicos e genéricos que possibilitam melhores interpretações de mudanças terapêuticas e mudanças relacionadas ao estado de saúde^15^.

A análise estatística por meio da verificação do TE foi relatada em um estudo^32^ que buscou verificar se o instrumento pesquisado era sensível a mudanças nas variáveis após uma intervenção. Com esse objetivo, ainda foram aplicados testes de correlações das mudanças entre as variáveis.

O desenho acima citado se assemelha ao do presente estudo e indica que foram seguidas recomendações quanto ao uso da estatística de TE, utilizada para análise da mudança na pontuação do CCVUQ-Br. No entanto, foram acrescentados os testes *t* de Student pareado e *z* de Wilcoxon para análise de uma mesma variável, sendo que essa escolha também não fugiu das recomendações anteriores, pois, em outros estudos, o teste *t* foi citado como a estatística mais utilizada para o cálculo da sensibilidade, em associação com o *z* de Wilcoxon^15^.

### Discussão dos resultados

As características da amostra estudada estão de acordo com pesquisas anteriores em portadores de úlcera venosa, inclusive pesquisas com o CCVUQ^5^^,^ 8.

Semelhanças são encontradas também em relação à ocupação dos portadores da úlcera, que na presente pesquisa encontravam-se na sua maioria aposentados (54,9%), sendo grande parte (25,5%) aposentados pela doença^33^.

Ao observar a redução dos valores da EVA dor e do tamanho da úlcera, pode-se observar que houve também melhora na QV do portador, identificada por uma diminuição das pontuações do CCVUQ-Br. A melhora da QV após conduta terapêutica ocorreu em outro estudo no qual, após 8 meses de tratamento com bota de Unna, houve melhora da QV^34^.

### Discussão dos resultados da responsividade

Para análise da significância das mudanças que ocorreram no CCVUQ-Br e em seus domínios, na EVA dor e no tamanho da úlcera, foram calculados o TE e o η^2^ parcial. Os resultados variaram de TE com sensibilidade baixa para um domínio do questionário a sensibilidade moderada e alta para os demais domínios e também para EVA dor e tamanho da úlcera. Essa variação em valores de TE, mesmo entre domínios de um mesmo questionário, também foi verificada no estudo de um instrumento para feridas^15^.

Na análise dos resultados das correlações das mudanças ocorridas no CCVUQ-Br com aquelas ocorridas em outras medidas de relevância clínica, as mais altas correlações ocorreram com a escala EVA dor e a classificação CEAP. Menores correlações ou correlações inexistentes foram notadas com as mudanças no tamanho da úlcera e na Escala de Avaliação Global de Mudança, fato que pode ser justificado por um estudo que comparou a QV de pacientes com úlceras ativas e úlceras cicatrizadas e concluiu que a cicatrização da úlcera não contribuiu para a melhora da QV dos pacientes^35^. A baixa correlação entre essas duas medidas possivelmente se deve ao fato de que seria necessária uma mudança maior relacionada às características das feridas.

## CONCLUSÃO

O questionário CCVUQ-Br, de QV para úlcera venosa, é sensível na análise longitudinal, quando utilizado na população brasileira.
